# The Effect of Perioperative Fluid Management on Intraocular Pressure during Gynecologic Laparoscopic Pelvic Surgery

**DOI:** 10.1155/2018/1457851

**Published:** 2018-03-15

**Authors:** Izakson Alexander, Sindawi Ahmad, Ben Shachar Inbar, Pikkel Joseph

**Affiliations:** ^1^Department of Anesthesiology, Ziv Medical Center, Zefat, Israel; ^2^Faculty of Medicine in the Galilee, Bar Ilan University, Zefat, Israel; ^3^Department of Gynecology & Obstetrics, Ziv Medical Center, Zefat, Israel; ^4^Department of Ophthalmology, Ziv Medical Center, Zefat, Israel

## Abstract

**Purpose:**

Visual loss is a devastating perioperative complication that can result from elevated intraocular pressure (IOP). The Trendelenburg position during surgery increases IOP. The purpose of this study was to quantify IOP changes in patients undergoing laparoscopic hysterectomy, at different time points and body positions throughout the procedure, and to compare fluctuations of IOP during the perioperative period according to two fluid management protocols.

**Methods:**

Thirty women scheduled to undergo elective gynecologic laparoscopic pelvic surgery were randomly allocated to receive a liberal or restrictive fluid management protocol. IOP, mean arterial pressure, heart rate, exhaled tidal volume, end-tidal CO_2_, and ocular perfusion pressure were assessed prior, during, and postsurgery, at 8 time points altogether.

**Results:**

Mean changes in IOP were similar for the two protocols; the peak IOP was at the steep (peak) Trendelenburg position. For each protocol, IOP correlated positively with mean arterial pressure, and mean blood pressure correlated with ocular perfusion pressure.

**Conclusion:**

IOP was elevated during laparoscopic pelvic surgery and particularly at the steep Trendelenburg position. No differences were found in any of the parameters examined according to a liberal or restrictive fluid management protocol.

## 1. Introduction

Visual loss is a devastating perioperative complication with an estimated incidence of 0.01–1%, depending on the type of surgery [1, 2]. Among the leading causes of this complication are retinal vascular occlusion, ischemic optic neuropathy, either anterior or posterior, and a rise in intraocular pressure that may cause vascular occlusion [3]. Recognized preoperative risk factors include hypertension, diabetes, polycythemia, smoking, renal failure, narrow-angle glaucoma, atherosclerotic vascular disease and collagen vascular disorders [4, 5]. Hypotension and anemia were reported as possible intraoperative risk factors for developing ischemic optic neuropathy [5].

Laparoscopic surgery is associated with several physiological changes that tend to increase IOP [6]. As for many surgical procedures, laparoscopic gynecological surgery requires specific body positioning in which the patient is placed in a steep Trendelenburg position (25–45 degrees head down). This positioning uses gravity to pull the abdominal viscera away from the operative field but is nonphysiologic and may have significant negative physiologic effects when maintained for long periods of time [7]. Serious ocular consequences, such as retinal detachment, have also been attributed to the Trendelenburg position [8]. While increased intraocular pressure (IOP) has been associated with this surgical positioning [9], the magnitude of this increase is unknown, particularly during long procedures and in combination with carbon dioxide insufflation during laparoscopy. Some clinicians consider surgery with a head-down position contraindicated in patients with ocular hypertension [9]. Evidence from research shows that elevated IOP can cause optic nerve ischemia by reducing blood flow to the eye [10]. Ocular perfusion pressure (OPP) is equal to mean arterial pressure (MAP) minus the IOP. Dramatic changes in OPP may overwhelm the autoregulation of blood flow to the eye [11]; however, the etiology of postoperative visual loss remains incompletely understood. The possible effect of perioperative fluid management on the outcome of surgical patients has recently been debated. A randomized multicenter trial found that a “liberal fluid” regime may result in overhydration, leading to deleterious effects on cardiac and pulmonary function, recovery of gastrointestinal motility, tissue oxygenation, wound healing, and coagulation [12]. The aim of our study was to quantify IOP changes in patients undergoing laparoscopic gynecologic pelvic surgery, at different time points and with changing body positions throughout the procedure, and to explore the effect of different protocols of perioperative fluid management on fluctuations in IOP during the perioperative period. This trial is registered with the registration number 003-12-ZIV at the Israeli Ministry of Health.

## 2. Materials and Methods

The study comprised 30 consecutive women who were scheduled for elective gynecologic laparoscopic pelvic surgery between January 1, 2015, and December 31, 2015, and who met study inclusion criteria. The exclusion criteria were age older than 70 years or less than 18 years, body weight > 150% of the ideal body weight, acute or chronic eye disease, use of any medication known to alter IOP, patients not capable or willing to sign informed consent. The study was approved by Helsinki Committee of Ziv Medical Center, and written informed consent was obtained from all patients prior to surgery.

Participants were alternately allocated to two equal groups (15 patients each), to receive either a liberal or restrictive fluid management protocol [13], based on ideal body weight (see Table 1). Demographic and anamnestic data were collected from all participants including age, body mass index (BMI), number of children, previous surgery, ethnic origin, and duration and type of surgery. The peak level of the Trendelenburg position was recorded for all patients. All participants were operated in the morning or early afternoon hours to avoid diurnal variations in IOP. All were premedicated with 5.0 mg of diazepam orally, 90 min before the induction of anesthesia. In both groups, anesthesia was induced with propofol 2.5 mg/kg and fentanyl 1.5 mg/kg was administered at induction. Rocuronium bromide 0.6 mg/kg was administered to facilitate tracheal intubation, when complete suppression of train-of-four twitches was achieved at the ulnar nerve. Further boluses of rocuronium 0.15 mg/kg were given as required, while maintaining 1-2 twitches of the train-of-four. Isoflurane and fentanyl concentrations were adjusted to maintain MAP within 20% of the preinduction value. After the intubation, the patients were put on a pressure control ventilation mode to achieve an expired tidal volume of 6–8 ml/kg. The minute volume was set to maintain end-tidal (ET) CO_2_ at 35–40 mmHg throughout the procedure. Pneumoperitoneum was created by intraperitoneal insufflation of CO_2_, with the patient in the supine position. Throughout the surgery, intraperitoneal pressure was maintained automatically at 14 mm Hg by a CO_2_ insufflator.

IOP was measured using a Tono-pen® XL by a trained ophthalmologist who was unaware of the perioperative fluid administration regimen. Measurements were repeated if the variability between sequential measurements exceeded 5%. Two sets of measurements for each eye were collected (two IOP readings per eye, each of which represents the average of a series of four measurements, as described previously). All measurements were performed by the same team of ophthalmologists. The depth of anesthesia was continuously evaluated with an entropy monitor. MAP, heart rate (HR), exhaled tidal volume, ET CO_2_, entropy values, IOP and OPP, were recorded at the following time points: T1—before the induction of anesthesia; T2—after the induction of anesthesia, in a supine and horizontal position, mechanically ventilated, before pneumoperitoneum; T3—after the pneumoperitoneum was established; T4—while the pneumoperitoneum was established, with a 15°–20° head-down tilt; T5—while the pneumoperitoneum was established, steep (peak) Trendelenburg position; T6—after the pneumoperitoneum was evacuated in the horizontal position; T7—in the recovery room, 30 degrees head up, 30 min after tracheal extubation; and T8—on the first postoperative day. Since blood pressure affects IOP, we examined the relationship between MAP and IOP at various concentrations of fluid intake in both study groups.

After each change in position and intraperitoneal pressure, a 5-minute period was allowed for stabilization before measurements were performed. Pneumoperitoneum was created by intraperitoneal insufflation of CO_2_ with the patient in the supine position. Throughout the surgery, intraperitoneal pressure was maintained automatically at 14 mm Hg by a CO_2_ insufflator.

### 2.1. Statistical Analyses

The *t*-test for the independent groups was used to examine the difference of IOP between the two treatments (liberal fluid group versus restrictive fluid group). Pearson's correlation was used to calculate correlations between the continuous variables and chi-square correlation between categorical variables. Values at *p* < 0.05 were considered statistically significant. The study was approved by the local bioethical committee of Ziv Medical Center, Israel.

## 3. Results

**Figure 1:**
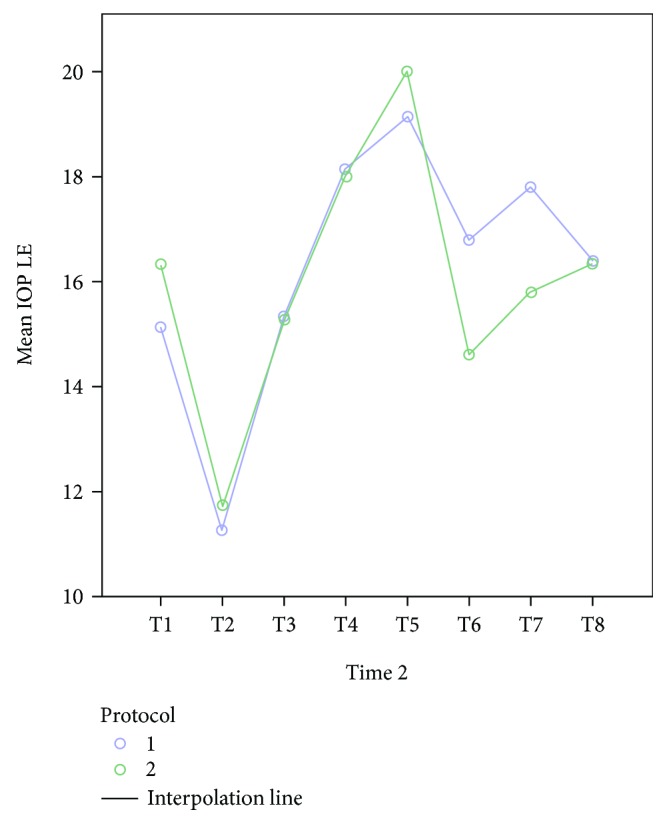
Mean changes of IOP in the left eye (LE) for the two fluid management protocols. T4 and T5 refer to the time points at which patients were in the Trendelenburg position with CO_2_ insufflation of the abdomen. 1: liberal fluid protocol. 2: restrictive fluid protocol.

**Figure 2:**
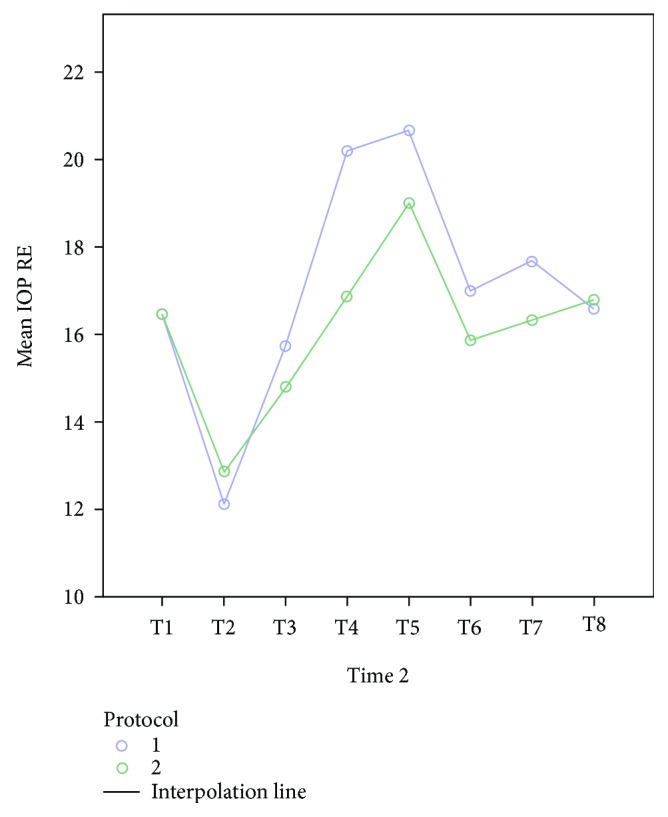
Mean changes of IOP in the right eye (RE) for the two fluid management protocols. T4 and T5 refer to the time points at which patients were in the Trendelenburg position with CO_2_ insufflation of the abdomen. 1: liberal fluid protocol. 2: restrictive fluid protocol.

**Figure 3:**
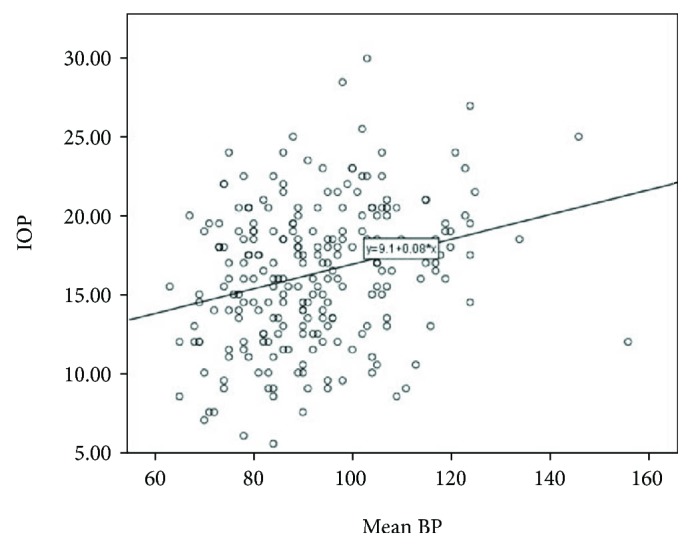
For both fluid protocols, the linear correlation between IOP and MAP did not differ at different concentrations of fluid intake (correlation is significant at the 0.01 level; *r* = 0.275; *p* value = 0.001).

**Figure 4:**
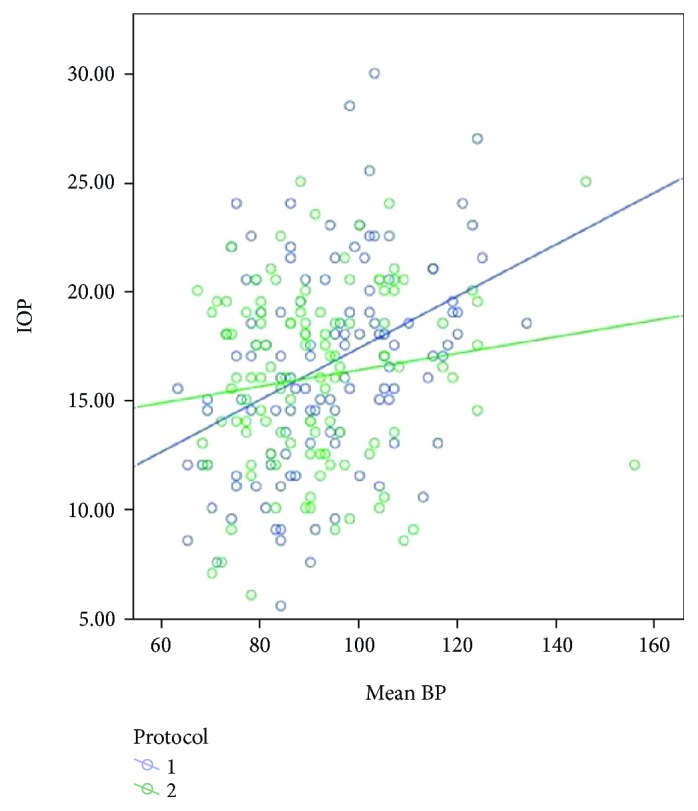
IOP versus MAP for each fluid protocol. 1: liberal fluid protocol. 2: restrictive fluid protocol.

**Figure 5:**
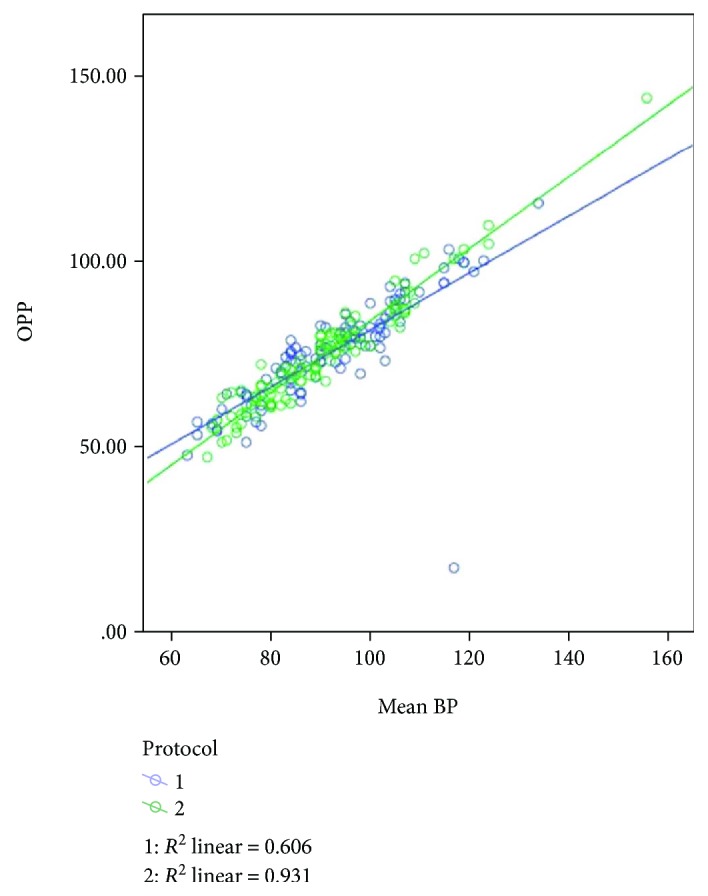
The correlation between OPP and MAP for both protocols is significant at the 0.01 level (*r* = 0.871, *p* = 0.001). OPP = MAP-IOP.

The mean age of the 30 patients was 40.7 years. Other than a gynecological problem that indicated surgery, they were all healthy. All patients underwent an uneventful surgery and were discharged from the hospital within 2-3 days after surgery. Mean changes in IOP were similar for the two fluid management protocols, for the left and right eyes (Figures 1 and 2, resp.). After the induction of anesthesia (T2), when the patient was in a supine and horizontal position, mechanically ventilated, before pneumoperitoneum, the mean IOP was significantly lower than the mean IOP before the anesthesia (T1): 16.07+ 3.45 versus 11.91+ 4.45 (*p* < 0.0001; data for all 30 patients). Subsequently, a far greater increase in IOP was observed during and immediately following Trendelenburg positioning (T4, T5, and T6). The peak IOP for both protocols was when pneumoperitoneum was established and the position was the steep (peak) Trendelenburg position (T5). On the first postoperative day (T8), the mean IOP for both groups was approximately equal to the mean preanesthetic IOP (T1). For both fluid management groups, the relationship between IOP and MAP did not change at different fluid intakes. Considering the data of all 30 participants, IOP correlated positively with MAP (Figure 3). Examination of the groups separately showed similar linear correlations (Figure 4). The correlation of MAP and OPP was similar in both groups (Figure 5).

As in general positioning did not have an effect on the IOP, we compared the durations of the Trendelenburg position between the 2 study groups. The mean time of this position in the liberal fluid group was 119 ± 21 minutes while in the restrictive fluid group 110 ± 17 minutes. This difference was not found to be statistically significant.

## 4. Discussion

The main findings of this prospective study were the following: (1) similar IOP measurements between patients who were treated with different preoperative fluid management protocols; (2) reduction in IOP following the induction of anesthesia in a supine and horizontal position and while patients were mechanically ventilated; (3) significant elevation of IOP during pneumoperitoneum and specifically in the steep Trendelenburg position; and (4) normal levels of IOP at about 12 hours postsurgery, similar to levels measured prior to the operation. Elevation of IOP may be problematic in patients who undergo gynecologic laparoscopic pelvic surgery and who also have chronic simple glaucoma. For patients with progressive damage to the optic disc, elevated IOP may exacerbate the injury to the retinal nerve fiber layer (RNFL) and further impair the visual field. In such patients, we suggest that a thorough ophthalmic examination, including a visual field test, the measurement of RNFL thickness and IOP, should be done prior to the gynecologic laparoscopic pelvic surgery. In extreme cases, gynecologists should consider another surgical approach, since, as shown in our study, the elevation of IOP in these surgeries is significant and the relatively long operating time raises the risk of damage to the RNFL. For measuring IOP, we used the Tono-pen due to its speed, ability to make measurements on multiple patients, ease of use including disposable latex tip covers, accuracy in a variety of positions, [14] reliability, and safety [14]. Though the effects of surgical positioning and laparoscopic procedures on IOP have been previously investigated [4, 5, 7], the current study focused specifically on the effects in gynecologic laparoscopic pelvic surgery and presented IOP measurements at several points of time. Visual loss after surgery is relatively rare [15]; however, preventing such devastating events is crucial. We suggest that a rise in IOP is one possible cause of this phenomenon. Gynecologic laparoscopic pelvic surgery is usually safe and has many advantages. Nevertheless, no procedure is 100% safe. We advise due attention to IOP fluctuations during these procedures, to avoid damage in patients who already have glaucoma optic problems. Though the number of participants is this study was relatively small, the statistical analyses showed a clear tendency of IOP elevation, as it was described previously.

## 5. Conclusion

Pneumoperitoneum, and specifically the steep Trendelenburg position, during gynecologic laparoscopic pelvic surgery may induce a significant elevation of IOP. This elevation was shown not to differ according to the protocol for perioperative fluid management. Surgeons who conduct such operations should consider this risk, especially in patients who already have glaucoma. Further studies are required to establish our findings.

## Figures and Tables

**Table 1:** Details of the two fluid protocols.

Time	Liberal fluid group	Restrictive fluid group
Before surgery	Fasting from midnight	Fasting from midnight
During surgery	7 ml/kg/hr RL during first intraoperative hr, 5 ml/kg/hr for the subsequent hours	RL according to “4-2-1” 4 ml/kg/hr for first 10 kg (40 ml/hr) Then 2 ml/kg/hr for next 10 kg (20 ml/hr) Then 1 ml/kg/hr for any kg over 20 kg of weight This always gives 60 ml/hr for first 20 kg; then you add 1 ml/kg/hr for each kg over 20 kg
After surgery (PACU)	1.5 ml/kg/hr	“4-2-1” rule
After operation at the ward day of surgery	1.5 ml/kg/hr RL	1.5 ml/kg/hr RL
Postoperative day 1	1.5 ml/kg/hr RL, oral fluids	1.5 ml/kg/hr RL, oral fluids
Postoperative day 2	Oral fluids and solid food according to surgical allowance	Oral fluids and solid food according to surgical allowance

RL: Ringer's lactate solution.
